# Variability in Single Digit Addition Problem-Solving Speed Over Time Identifies Typical, Delay and Deficit Math Pathways

**DOI:** 10.3389/fpsyg.2018.01498

**Published:** 2018-08-14

**Authors:** Robert A. Reeve, Sarah A. Gray, Brian L. Butterworth, Jacob M. Paul

**Affiliations:** ^1^Melbourne School of Psychological Sciences, University of Melbourne, Melbourne, VIC, Australia; ^2^Centre for Educational Neuroscience, University College London, London, United Kingdom

**Keywords:** typical, delayed, deficit math pathways, single digit addition problem solving speed variability, subitizing ability, longitudinal analysis

## Abstract

We assessed the degree to which the variability in the time children took to solve single digit addition (SDA) problems longitudinally, predicted their ability to solve more complex mental addition problems. Beginning at 5 years, 164 children completed a 12-item SDA test on four occasions over 6 years. We also assessed their (1) digit span, visuospatial working memory, and non-verbal IQ, and (2) the speed with which they named single numbers and letters, as well the speed enumerating one to three dots as a measure of subitizing ability. Children completed a double-digit mental addition test at the end of the study. We conducted a latent profile analysis to determine if there were different SDA problem solving response time (PRT) variability patterns across the four test occasions, which yielded three distinct PRT variability patterns. In one pattern, labeled a typical acquisition pathway, mean PRTs were relatively low and PRT variability diminished over time. In a second pattern, label a delayed pathway, mean PRT and variability was high initially but diminished over time. In a third pattern, labeled a deficit pathway, mean PRT and variability remained relatively high throughout the study. We investigated the degree to which the three SDA PRT variability pathways were associated with (1) different cognitive ability measures, and (2) double-digit mental addition abilities. The deficit pathway differed from the typical and delayed pathway on the subitizing measure only, but not other measures; and the latter two pathways also differed from each other on the subitizing but not other measures. Double-digit mental addition problem solving success differed between each of the three pathways, and mean PRT variability differed between the typical and the delayed and deficit pathways. The latter two pathways did not differ from each other. The findings emphasize the value of examining individual differences in problem-solving PRT variability longitudinally as an index of math ability, and highlight the important of subitizing ability as a diagnostic index of math ability/difficulties.

## Introduction

One goal of early math instruction is to help children acquire the basic arithmetic skills necessary to solve more complex calculation problems. Ensuring children acquire good single digit addition (SDA) number fact abilities, for example, is a learning objective in many countries (OECD, 2014). While instructional emphases differ (e.g., from a focus on rote learning to reasoning strategies), children tend to use so-called procedural strategies (e.g., counting all items) before so-called conceptual strategies (e.g., decomposition of number facts) to solve SDA problems (Butterworth, 2005; Geary and Hoard, 2005; Siegler, 2016); and, children may use both procedural and conceptual strategies on a single test occasion. While the association between the strategies used to solve SDA problems and problem-solving success varies within and across age, most children solve SDA problems eventually (Paul and Reeve, 2016). Nevertheless, this acquisition variability raises the possibility that different SDA acquisition pathways are embedded within a general acquisition pathway. Insofar as different SDA acquisition pathways can be identified, it is possible they lead to a single ability end-point (equifinality); it is also possible that different pathways reflect different ability profiles, which would have implications for our understanding of math development.

The present study addressed the issue of whether it is possible to distinguish typical, deficit and delay SDA acquisition pathways in primary-aged school children based on changes in the variability of the speed with which children solved SDA problems over a 5-year period. We focused on variability in SDA problem-solving speed because arguably it represents an index of changes in SDA problem-solving efficiency, especially when examined over time. Focusing on problem solving speed also allowed us to examine SDA problem solving after children were able to solve problems correctly. In general, it would be expected that children’s SDA problem-solving speed trajectory would decline and become less variable over time. It is possible that problem-solving speed will decline slowly for some children (a delayed pathway?), or continues to be variable (a deficit pathway?) over time. Given the importance of SDA abilities in curricula, understanding the factors associated with different SDA developmental pathways may have diagnostic significance, as well as contribute insights to our understanding of the nature individual differences in math development more generally.

### SDA Strategy Change

The strategies children employ to solve single addition problems skills, on average, change in their conceptual sophistication over time and are claimed to represent changes in math reasoning abilities (Baroody, 2003; Butterworth, 2005; Siegler, 2006; Geary et al., 2007; Jordan et al., 2009; Paul and Reeve, 2016). Children initially guess answers, following which they may use a *count all* strategy to individually enumerate the numbers of the two addends. Subsequently, they may adopt a *count on* strategy (specifying the cardinal value of the first addend, and sequentially enumerating the numbers of the second addend). Children may then employ a *min* strategy (counting on from the larger of the two addends when it is the second term). In time, they begin using more sophisticated strategies, including the *decomposition* of number facts and *retrieval* of answers from memory (Baroody and Dowker, 2003; Geary et al., 2007).

How should these changes in the acquisition of SDA problem solving abilities be characterized? As Siegler notes, the development of children’s reasoning strategies is more variable than often acknowledged (Siegler, 2007, 2016). Siegler (1996, 2000) characterized reasoning development in terms of changes in the selection of strategy options over time. Commonly, children use a mix of strategies to solve problems, with a progressive reduction of less sophisticated strategies accompanying the acquisition of problem solving ability (usually across age). That is, with age and/or experience, children solve problems more quickly and select more efficient strategies, and less efficient strategies disappear from their repertoire (Torbeyns et al., 2004).

Is strategy change the same for all children, or are there different strategy change profiles and, if there are, what do they imply about children’s abilities? Siegler’s overlapping wave model suggests the acquisition of problem solving competence may be analyzed along five dimension of change—path, rate, breadth, sources and variability (see Siegler, 2005). Siegler and colleagues (Siegler, 2005, 2016) suggest these dimensions may be studied using the so-called microgenetic method in which multiple observations of strategy change are made from the beginning of change to the point at which strategy-use becomes relatively stable. Strategies are subjected to a trial-by-trial analysis, the aim of which is to infer the processes that give rise to strategy change (Siegler and Crowley, 1991). While the focus on microgenetic methods hints at the multidimensional nature of individual differences in the acquisition of a specific ability, it has had relatively little to say about (1) the significance of different acquisition pathways, (2) the cognitive indices associated with different pathways, or (3) whether the same indices are relevant at different change points.

In the present study, we investigated changes in SDA problem solving speed variability (PRT) patterns longitudinally. The rationale for focusing on problem solving speed variability is we have found a close association between strategy-use and problem solving speed (Canobi et al., 1998, 2002; Paul and Reeve, 2016; Major et al., 2017). For example, a *count all* strategy, where each addend is individually enumerated, takes more time to execute and is more error prone than a *retrieval* strategy where answers are retrieved from memory (i.e., the answer is known and does not require computation). And, we have found a strategy-speed correlation independent of whether SDA problem was solved correctly or not (Paul and Reeve, 2016). We argue that the time taken for an individual to answer to a SDA problem is a defensible proxy for SDA strategy use (Major et al., 2017). Moreover, we can analyze the variability in SDA PRTs after individuals have learned to solve problems correctly.

Analyzing the variability in the speed with which individuals react to an event or solve problems has a long history in research on the neurophysiological basis of individual differences (Jensen, 1992). Indeed, it was pointed out 50 years ago that inter-event variability in RTs is not necessarily a measurement error in the narrow sense, but maybe a robust phenomenon in which there are reliable individual differences in RT patterns (Berkson and Baumeister, 1967). Recent research examining the RT patterns of children with ADHD, for example, shows they tend to have atypical RT patterns on attention tasks (Lewis et al., 2017). However, as far as we are aware, no research has investigated the significance of different RT patterns in SDA problem solving.

### Cognitive Factors That May Affect SDA Strategies

A number of studies have investigated the association between cognitive factors (e.g., IQ, working memory), SDA strategy-use and problem solving ability (e.g., Paul and Reeve, 2016). Interpreting the importance of age-related factors responsible for general SDA abilities can be problematic since many abilities are correlated with age (Reeve et al., 2015). Furthermore, correlations tend to be modest, suggesting significant within-age variability in the factors affecting math abilities (Dowker, 2005). Nevertheless, associations have been found between SDA problem solving abilities, and some cognitive competencies (i.e., IQ, working memory) as well as core number abilities (dot enumeration, magnitude comparison) (Paul and Reeve, 2016). Increases in working-memory span (WM), for example, are associated with SDA problem solving accuracy (Raghubar et al., 2010). And poor WM capacity is thought to affect SDA strategies (e.g., by affecting the ability to monitor counting: see Dowker, 2005), and good WM capacity is associated with sophisticated SDA strategies (Geary, 2011; Geary et al., 2012). However, the association between the form of WM and math ability changes with age. In the young, math abilities tend to be correlated with visuospatial working memory (VSWM); and in older children verbal WM is more associated with math ability (De Smedt et al., 2009; Ashkenazi et al., 2013; van der Ven et al., 2013). This finding is consistent with the claim that visuospatial reasoning abilities are critical for early math (Gelman and Butterworth, 2005; Siegler and Mu, 2008; Dehaene and Brannon, 2011; Reeve et al., 2015).

In some studies non-verbal intelligence (NVIQ) is related to math abilities (Szűcs et al., 2014; however, see Reeve et al., 2012). In a longitudinal study Geary et al. (2017) reported that NVIQ was a stable predictor of children’s math achievement (see also Van de Weijer-Bergsma et al., 2015; Lee and Bull, 2016; Tolar et al., 2016). One explanation for this association is NVIQ, in part, requires visuo-spatial abilities which are thought to be necessary for early math problem-solving ability (Szűcs et al., 2014). The question of the kinds of visuo-spatial skills that support different kinds of early math abilities is yet to be resolved, however.

Core number abilities are claimed to support early math development (Butterworth, 2010). The ability to rapidly and precisely enumerate small sets, for example, predicts concurrent and future math achievement (Reeve et al., 2012; Sasanguie et al., 2013; Bartelet et al., 2014; Gray and Reeve, 2014; Major et al., 2017). Dot enumeration tasks assess at least two components: a subitizing and a counting component. Subitizing is assessed by evaluating the way small sets (*n* < 4) are enumerated, which is usually accurately, rapidly and without error; counting is evaluated by assessing the way larger sets (*n* > 4) are enumerated, which usually more slowly and prone to counting errors (Schleifer and Landerl, 2011).

Reeve et al. (2012) identified three distinct dot enumeration profiles in 5-year-olds and showed profile membership remained stable over the primary school years. The three profiles differed in subitizing range, subitizing slope and intercept, but not counting slopes. Moreover, the profiles were associated with differences in math problems solving abilities. A similar pattern of findings has been observed in preschool children (Gray and Reeve, 2014, 2016). We suggest that children with limited subitizing abilities may lack the ability to readily extract pattern or grouping information from small sets of dots (Butterworth, 2003; Ashkenazi et al., 2013). Why might this be important for numerical cognition? The ability to “know” the number “2” or “3” can be represented by a collection of two or three dots respectively, without counting individual dots, is an index of set knowledge (Butterworth, 2010); and set manipulation represents an important aspect of the development of numerical cognition (Gallistel and Gelman, 1992). The degree to which set knowledge changes in childhood is yet to be specified, however.

In recent research, Major et al. (2017) showed that dot enumeration profiles, in conjunction with performance on a standardized math test (the TEMA), assessed at school entry, predicted children’s SDA problem solving speed longitudinally. However, the Major et al. findings were based on a general longitudinal path analytic model and their findings are silent about the possibility of different SDA PRT pathways, which is the focus of the current research.

### The Current Research

The current research examined changes in children’s SDA problem-solving response time variabilty (PRT) four times over 6 years (at 6, 7, 9, and 10 years) to determine whether it is possible to identify separate SDA PRT trajectories across time. Insofar as different speed trajectories could be identified, we investigated the degree to which different cognitive indices (i.e., VSWM assessed at 7 years, WM assessed at 9 years, speed naming numbers/letters, non-verbal IQ, and dot enumeration RTs in the subitizing range assessed at 9 years) were associated with different SDA PRT pathways; and the degree to which different SDA PRT pathways predicted performance on a double-digit mental addition (DDA assessed at 10 years) accounting for other cognitive abilities.

We included the VSWM and WM measures because math abilities tend to be correlated with VSWM in young children and with verbal WM in older children (Ashkenazi et al., 2013). We included the DDA task because on face-value it is a conceptually more complex version of the SDA task (see Major et al., 2017; Lemaire and Brun, 2018). Of interest is the degree to which different math acquisition pathways (i.e., variability in single digit addition problem speed over time) are associated with a common outcome. We included the naming numbers/letters speed task to assess for the possibility that findings reflect the speed with which information, particularly numerical information, is retrieved from memory.

We included the dot enumeration measure since previous research had shown that differences in responding to 1–3 dots is associated with math abilities at school entry and over the long term (Reeve et al., 2012; Major et al., 2017).

To identify different possible SDA speed variability trajectories over time, we used latent profile analysis (LPA) based on each individual child’s mean variability in SDA PRTs at each of the four SDA assessment times. In LPA individuals are assigned to one of a number of subgroups or profiles that share common data patterns (Van Der Maas and Straatemeier, 2008). (This form of analysis has been used to characterize changes in the relationship between SDA strategy over time and VSWM—see Geary et al., 2009.)

Given the analytic focus of our research is change in the variability in SDA PRTs over time (rather than SDA strategy-use or problem-solving success), it would seem a priori reasonable to expect at least three PRT profiles to emerge from LPA: (1) a typical pathway in which mean SDA PRT variability diminishes over time; (2) a delayed SDA PRT pathway in which PRTs variability is high initially, but diminishes over time; and (3) a deficit pathway in which PRT variability remains relatively high over time. We acknowledge other profiles may emerge from LPA; however, we cannot anticipate what these might be a priori.

Insofar as SDA PRT variability pathways reflect different math specific (dot enumeration—subitizing ability, speed naming numbers) and/or general cognitive abilities (VSWM, WM, speed naming letters and NVIQ), we test several working hypotheses. Specifically, we expected children assigned to a delayed SDA PRT pathway would differ from children assigned to a typical pathway in their general cognitive capacities, but not their math specific ability (subitizing ability). Given the SDA PRTs of children in the delayed profile approach that of children in the typical profile over time, the delay is likely attributable to differences in general cognitive abilities. We expected children assigned to a deficit SDA PRT profile would differ from children in the typical and delayed pathway in their subitizing ability, and possibly their general cognitive abilities. This hypothesis is based on previous research which shows children with a math deficit also have poor subitizing abilities, but not necessarily general cognitive difficulties (Reeve et al., 2012; however see Gray and Reeve, 2016).

Insofar as different SDA PRT variability pathways reflect different arithmetic abilities, children assigned to the typical profile would be expected to perform better (would show less variability in response time and be more accurate) than those assigned to the delay profile who, in turn, would perform better than children assigned to a deficit profile on the double-digit mental addition task (DDA).

## Materials and Methods

### Participants

One hundred-sixty-four children (*M* = 72.59 months, *SD* = 4.58 months at the beginning of the study), comprising 65 girls (*M* = 71.52 months, *SD* = 4.47 months) and 99 boys (*M* = 73.29 months, *SD* = 4.54 months), attending schools in middle-class suburbs of a large Australian city, participated in the study. All children spoke fluent English, had normal or corrected to normal vision and had no known learning disabilities (according to school personnel). The data reported herein were collected on four different occasions, namely, when children were 6, 7, 9, and 10 years of age. The children were part of a larger study investigating the development of math ability in preadolescent children across the primary/elementary school years (see Reeve et al., 2012 for details—note, only children who completed all assessments were included in the present study). At Time 2 children were 7-years-old (*M* = 85.59 months, *SD* = 4.08 months), at Time 3 children were 10-years-old (*M* = 122.85 months, *SD* = 4.26 months), and at Time 4 children were 11-years-old (*M* = 129.49 months, *SD* = 4.55 months). The study was conducted in compliance with the requirements of the authors’ University’s Human Ethics Committee and the agreement of participating schools. Parents provided written consent allowing their child to participate in the project.

### Materials and Procedure

#### Single-Digit Addition (Completed on All Four Occasions)

Twelve SDA problems were presented at each time point (see **Table 1**). Each pair of digits was presented in both orders (i.e., 2 + 5 and 5 + 2) to counterbalance and allow for the possibility to solve problems using a “min-counting” strategy (e.g., begin the count sequence from the largest addend to minimize the counting distance, irrespective of the fact that problems are read from left to right: see Paul and Reeve, 2016). Before beginning the task, children completed practice trials to familiarize them with the requirement to solve problems as quickly and as accurately as possible. Problems were presented in a random order. Problems appeared in the center of a 15″ laptop screen in the form of a + b = . Problem-solving accuracy and response times were recorded. The Chronbach’s alphas, and associated 95% confidence interval for each SDA time measure, were—Time 1: 0.88 (0.85, 0.90); Time 2: 0.90 (0.87, 0.92); Time 3: 0.89 (0.86, 0.91); Time 4: 0.89 (0.87, 0.92).

**Table 1:** Single-digit addition problem set repeated across Time 1 – Time 4.

Pair	Left addend	Right addend	Total sum	Numerical distance
Pair 1	2	4	6	2
Pair 2	4	2	6	2
Pair 3	2	5	7	3
Pair 4	5	2	7	3
Pair 5	3	5	8	2
Pair 6	5	3	8	2
Pair 7	2	6	8	4
Pair 8	6	2	8	4
Pair 9	3	6	9	3
Pair 10	6	3	9	3
Pair 11	2	7	9	5
Pair 12	7	2	9	5

#### Double-Digit Addition (Completed at 10 Years)

Twenty-four pairs of double-digit addend problems were presented (e.g., 28 + 19), in which the sum of the addends was less than 100 (see **Table 2**). Problem-solving accuracy and response times were recorded (Cronbach’s alpha = 0.95: 95% CI = 0.94 – 0.96.)

**Table 2:** Double-digit addition problems.

23 + 16	24 + 18	16 + 27
52 + 34	29 + 53	47 + 38
46 + 37	25 + 12	37 + 46
12 + 25	46 + 53	42 + 35
18 + 24	38 + 47	53 + 29
34 + 52	19 + 28	15 + 31
31 + 15	16 + 23	27 + 16
53 + 46	28 + 19	35 + 42

#### Forward Corsi Span (Completed at 6 Years)

The Corsi Blocks task (Milner, 1971) assessed visuo-spatial working memory, and was administered and scored following Kessels et al. (2000) procedure. An interviewer taps a sequence of blocks that attempts to repeat: beginning with two blocks, increasing by one block following each correct reproduction, up to a maximum of nine blocks. Testing concluded after two failed trials. The longest correct block tap sequence is the VSWM span. Reliability was α = 0.70.

#### Backward Digit Span (Completed at 7 Years)

The backward version of the WISC-R Digit Span test was administered and scored as per the WISC-R Manual (Wechsler, 1986). This measure has been used to index WM capacity for verbal information (Geary et al., 2012). Reliability was α = 0.63.

#### Naming Numbers Naming Letters (Completed at 9 Years)

In the naming numbers and naming letters tasks, the numbers 1–9 and the letters A–J (excluding the letter I because of its similarity to the number 1), respectively, were used. The two tasks comprised 36 trials, four each for the nine stimuli. The stimuli for both tasks, all of which were approximately 2 cm high on screen, were presented in one of four fixed random orders; the only constraint was that each stimulus should be different to the immediately preceding stimulus. Presentation order of the naming numbers and naming letters tasks was counterbalanced. (Cronbach’s alphas: Naming Numbers = 0.96: 95% CI = 0.96 – 0.9; Naming Letters = 0.99: 95% CI = 0.99 – 0.99.)

#### Raven’s Colored Progressive Matrices (RCPM) (Completed at 9 Years)

The RCPM is a measure of non-verbal IQ suitable for young children. It was included to assess the association between SDA processing speed and intelligence (Luwel et al., 2013). RCPM was administered following manual instructions (Raven et al., 1986), and scored using age norms (Raven et al., 1998). Research show good inter-item consistency and split-half reliability in a sample of Australian children (Cotton et al., 2005). The reliability estimate for the current sample was good (α = 0.82).

#### Dot Enumeration (Completed at 10 Years)

Dot arrays comprising one to nine black dots (0.2 cm diameter) were presented on a white background. Dots were randomly positioned within a 15 cm × 11 cm grid and were no less than 2 cm apart (to reduce perceptual grouping cues). Each dot numerosity was presented eight times (*n* = 72 trials overall). Children were instructed to report as quickly and accurately as possible the number of dots in the array. Response accuracy and RTs were recorded. Here, only responses to dot arrays in the subitizing range (1–3 dots) were included in the analysis (24 trials). Previous research has shown differences in responding to 1–3 dots (i.e., differences in RTs, slope and intercept of the subitizing range) is associated with math abilities at school entry and over the long term (Reeve et al., 2012; Major et al., 2017). However, the speed enumerating dots in the counting range (5–8 dots) was not associated with math ability (Reeve et al., 2012). It is worth noting that Anobile et al. (2016) showed that numerosity, but not texture-density, discrimination correlates with math ability in children. (Cronbach’s alpha = 0.83: 95% CI = 0.79 – 0.86.)

### Rationale for Measures

We calculated a measure of SDA problem-solving RT variability (SDA_var_) for each child (*i*) by subtracting their average RT (*μ_i_*) at each time point from each of the twelve SDA problems (*q_j_*), and then taking the sum of the absolute values of these deviations (|*μ – q |*):

$$\begin{array}{c} {\mathrm{SDA}}_{\mathrm{var}}=\sum_{i=1}^{n}|q_{i}-\mu_{i}| \end{array}$$

The same procedure was used to create RT variability measures for the naming numbers RT_var_ (nine trials), naming letters RT_var_ (nine trials), dot enumeration (DE_var_, 24 trials), and double-digit addition (DDA_var_, 24 problems) tasks. For dot enumeration, only responses to dot arrays in the subitizing range (1–3 dots) were included in this analysis (24 trials). Previous research has shown differences in responding to 1–3 dots (i.e., differences in RTs, slope and intercept of the subitizing range) is associated with math abilities at school entry and over the long term (Reeve et al., 2012; Major et al., 2017).

Corsi span (VSWM) scores represent the average of two trials of the forward version of the task (see Kessels et al., 2000). Digit span scores were measured as the sum of the forward and backward versions of the WISC-R test. Raven’s (NVIQ) raw scores are used in analyses since scaled percentile scores were at ceiling level and non-normally distributed.

#### Analytic Approach

We used MPlus (Muthén and Muthén, 1998–2013) latent class/profile analysis to identify SDA problem-solving speed profiles. (It should be noted that we did not examine SDA problem solving success – most children were performing at ceiling on the second test occasions.) We estimated three LPA models with an increasing number of profiles based on expected patterns of change in SDA problem-solving variability over time: (1) a two profile solution would differentiate a typical pathway (e.g., decreased variability over time) from a deficit pathway (e.g., minimal decrease in variability over time); (2) a three profile solution would differentiate a typical pathway, a delayed pathway (e.g., slower decrease in variability over time compared to typical performance) and a deficit pathway; and (3) a four profile solution was expected to identify a typical pathway, a delayed pathway and a deficit pathway, while also allowing for the possibility of another different pathway (e.g., irregular shifts in variability over time).

Once profiles were identified, children were allocated to the profile with the highest probability of membership. To further distinguish between these pathways, One-way ANOVAs were conducted to characterize differences in measures of cognitive ability (i.e., VSWM, naming numbers RT variability, naming letter RT variability, digit span, NVIQ, and subitizing RT variability) between the profiles. One-way ANOVAs were also conducted to determine whether profiles were associated with double-digit addition problem-solving accuracy and response time variability. Regression analyses were conducted to determine the independent contribution of the profiles and cognitive abilities in predicting double-digit addition problem-solving accuracy and response time variability.

## Results

### Descriptive Statistics

Bivariate correlations and means (standard deviations) for measures are reported in **Table 3**. Of note, SDA PRT variability (SDA_var_ 6, 7, 9, and 10 years) showed an average decrease in PRT over time; however, there was significant variation in means over time, suggesting different patterns of variance may be embedded within the overall variance. We used LPA to investigate this possibility.

**Table 3:** Bivariate correlations and means (standard deviations) for all measures.

	1	2	3	4	5	6	7	8	9	10	11	12
(1) SDA_var_ 6 years	–											
(2) SDA_var_ 7 years	0.17^∗^	–										
(3) SDA_var_ 9 years	0.09	0.33^∗∗∗^	–									
(4) SDA_var_ 10 years	0.09	0.36^∗∗∗^	0.65^∗∗∗^	–								
(5) VSWM	–0.09	–0.07	–0.06	–0.18^∗^	–							
(6) Naming numbers RT_var_	–0.04	0.01	0.07	–0.03	–0.05	–						
(7) Naming letters RT_var_	–0.04	0.05	0.09	–0.01	–0.08	0.84^∗∗^	–					
(8) Digit span	–0.14	–0.12	–0.03	–0.03	0.05	0.09	0.08	–				
(9) NVIQ	0.08	–0.13	–0.06	–0.04	0.23^∗∗^	–0.11	–0.15	0.28^∗∗^	–			
(10) DE_var_	–0.08	0.29^∗∗^	0.45^∗∗^	0.55^∗∗^	–0.14	0.05	0.09	0.00	–0.09	–		
(11) DDA_acc_	–0.27^∗∗^	–0.26^∗∗^	–0.34^∗∗^	–0.27^∗∗^	0.04	–0.03	–0.07	0.12	0.01	–0.29^∗∗^	–	
(12) DDA_var_	0.25^∗∗^	0.42^∗∗^	0.40^∗∗^	0.58^∗∗^	–0.05	–0.03	–0.02	–0.15	–0.00	0.43^∗∗^	–0.52^∗∗^	–
Mean	39.99	23.92	8.93	7.89	3.66	3.38	4.44	9.13	31.64	4.81	0.86	75.32
*SD*	27.47	16.79	8.29	6.34	0.65	0.83	1.31	2.19	3.51	2.22	0.15	42.53

*SDA_*var*_ is the sum of trial-by-trial variability in single-digit addition problem-solving (12 trials); VSWM is the average block length across two forward trials; Naming numbers_*var*_ and Naming letters_*var*_ is the sum of trial-by-trial variability (nine trials); Digit span is the sum of sequence length across forward and backward trials; NVIQ is the raw Raven’s score; DE_*var*_ is the sum of trial-by-trial variability (24 trials); DDA_*acc*_ is the accuracy of double-digit addition problem-solving; and DDA_*acc*_ is the sum of trial-by-trial variability in double-digit addition problem-solving (24 trials). ^∗∗∗^*p* < 0.001; ^∗∗^*p* < 0.01; ^∗^*p* < 0.05.*

### SDA Problem Solving Speed Profiles

Latent profile models with two to four profiles were compared in terms of different goodness-of-fit indices to determine the best-fitting solution to the data. **Table 4** shows all relative fit statistics (AIC, BIC and aBIC) improved for models with an increasing number of profiles and entropy values were high (≥0.8, suggesting good separation of profiles; Clark and Muthén, 2009). While the four-profile solution provided better fit than the three-profile solution (i.e., significant bootstrap likelihood-ratio test scores; see **Table 4**), examination of the four profiles revealed two profiles were similar—both profiles showed patterns of delayed decrease in variability over time, which were not meaningfully different from each other. The three-profile solution characterized more distinct patterns of change in variability over time, and were more consistent with typical, delayed and deficit pathways. Since the three-profile model was a more parsimonious description of the data and was less likely to lead to over-fitting our sample than the four-profile model, the three-profile model was selected for further examination (see **Supplementary Material**).

**Table 4:** Latent profile analysis goodness-of-fit indices.

Profiles	Parameters	LL	AIC	BIC	aBIC	BLRT	Entropy
2	17	–2425.42	4884.83	4937.53	4883.71	<0.001	0.86
3	26	–2373.91	4799.83	4880.42	4798.11	<0.001	0.83
4	35	–2342.39	4754.78	4863.28	4752.47	<0.001	0.85

*LL, Log-likelihood; AIC, Akaike Information Criterion; BIC, Bayesian Information Criterion; aBIC, Adjusted Bayesian Information Criterion; BLRT, Bootstrap Likelihood-Ratio Test (100 draws).*

**FIGURE 1:**
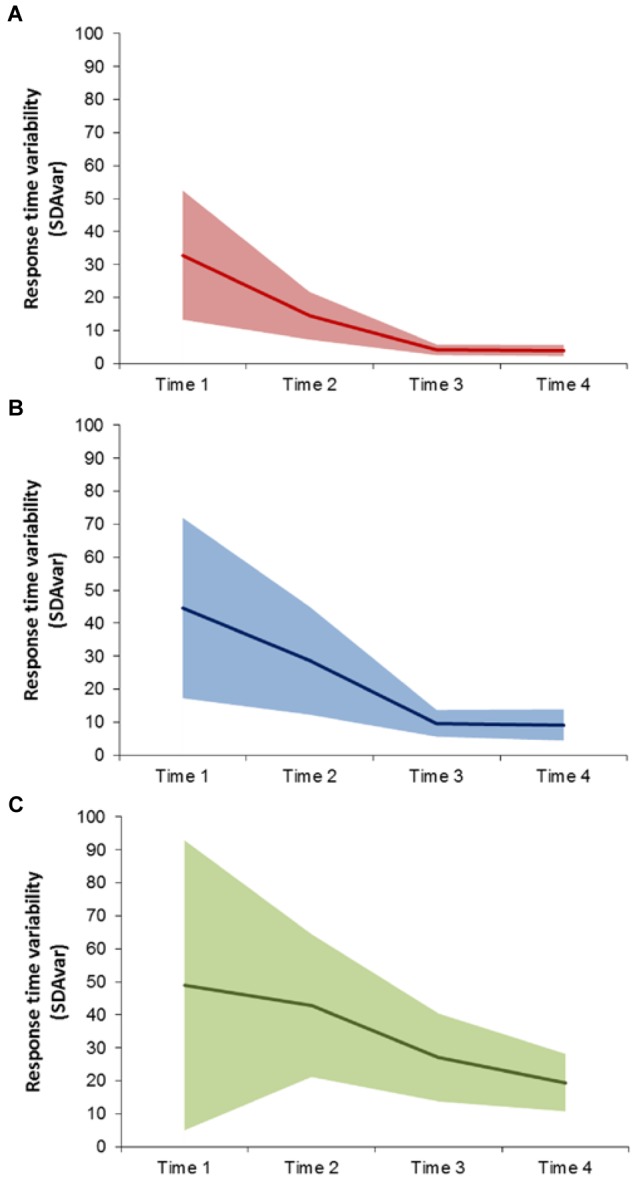
Three profiles identified from latent profile analysis of SDA problem solving response time variability (SDA_var_) assessed at 6-, 7-, 9, and 10-years **(A)** Typical, **(B)** Delayed and **(C)** Deficit pathways. Solid line represents profile mean SDA_var_ and shaded region represents ± 1 standard deviation.

The three profiles differed in mean RT and SDA variability measures over time (see **Figure 1**). The first pathway (Typical pathway, *n* = 71, 43.3%) showed a decrease in SDA problem-solving speed variability over time, and exhibited minimal variability at Times 3 and 4. The second pathway (Delayed pathway, *n* = 78, 47.6%) showed a similar decrease in RT variability over time; however, the variability was still decreasing at Times 3 and 4. The third pathway (Deficit pathway, *n* = 15, 9.1%) showed a decrease in RT over time but SDA variability remained high.

### Analysis of Cognitive Abilities Across SDA PRT Profiles

One-way ANOVAs were conducted to determine whether the measures of cognitive ability differed across the three profiles. Bias-corrected and accelerated bootstrap estimates (95% confidence, 1000 draws) are reported to account for unequal variance between profiles, and Welch correction for robust test of equality of means was applied when necessary. The profiles differed significantly in terms of subitizing RT_var_ [*F_WELCH_* (2, 34.93) = 8.48, *p* = 0.001, Levine = 17.37, *p* < 0.001]. The Typical pathway had significantly lower subitizing RT_var_ compared to the Delayed (*p* = 0.020) and Deficit (*p* = 0.011) pathways, while the Delayed pathway had significantly lower subitizing RT_var_ compared to the Deficit (*p* = 0.046) pathway. The pathways did not significantly differ in terms of VSWM span [*F*(2,161) = 0.89, *p* = 0.413], naming numbers RT_var_ [*F*(2,161) = 0.62, *p* = 0.540] or naming letters RT_var_ [*F*(2,161) = 0.59, *p* = 0.553], digit span [*F*(2,161) = 1.96, *p* = 0.144] or NVIQ [*F*(2,161) = 1.49, *p* = 0.228].

### Association Between Variability Pathways and Double Digit Addition Ability

A one-way ANOVA (bias-corrected and accelerated bootstrap estimates) compared the double-digit problem-solving accuracy across the three profiles. Double-digit accuracy differed significantly between the profiles [*F_WELCH_*(2,35.22) = 13.12, *p* < 0.001, Levine = 19.97, *p* < 0.001]. Post hoc comparisons (corrected for unequal variances, Games-Howell) showed the *Typical Pathway* (*M* = 0.92, *SD* = 0.09) had significantly higher double-digit problem-solving accuracy than the *Delayed Pathway* (*p* < 0.001) and *Deficit Pathway* (*p* = 0.016); the *Deficit Pathway* (*M* = 0.75, *SD* = 0.20) had the lowest double-digit problem-solving accuracy, but was not significantly different from the *Delayed Pathway* (*M* = 0.83, *SD* = 0.16).

A separate one-way ANOVA compared response time variability between profiles, which showed double-digit response time variability differed significantly across profiles [*F_WELCH_*(2,35.65) = 25.10, *p* < 0.001, Levine = 9.67, *p* < 0.001]. Post-hoc comparisons showed the *Typical Pathway* (*M* = 53.55, *SD* = 27.12) had significantly lower double-digit response time variability than both *Delayed Pathway* (*p* = 0.001) and *Deficit Pathway* (*p* < 0.001); the *Delayed Pathway* (*M* = 85.20, *SD* = 38.93) had significantly lower double-digit response time variability than the *Deficit Pathway* (*p* = 0.037); and the *Deficit Pathway* (*M* = 126.96, *SD* = 56.89) had the highest double-digit response time variability.

The cognitive abilities and pathway membership (dummy coded relative to the *Deficit Pathway*) were entered into separate linear regression analyses to determine the degree to which they predicted double-digit addition problem-solving accuracy (Model 1, **Table 5**) and response time variability (Model 2, **Table 6**). (Note, we report separate analyses that included/exclude the pathways for clarify sake.) Overall, only the subitizing measure significantly predicted DDA accuracy [Model 1a (with cognitive abilities): *F*(6,157) = 3.19, *p* = 0.006; Model 1b (with variability profiles): *F*(8,155) = 4.42, *p* < 0.001] and response time variability [Model 2a (with cognitive abilities): *F*(6,157) = 7.16, *p* < 0.001]; Model 2b (with variability profiles): *F*(8,155) = 10.55, *p* < 0.001).

**Table 5:** Model 1: Linear regressions predicting DDA PRT success.

	*Beta*	*SE*	*β*	*t*	*p*
**Model 1a – Cognitive Abilities**					
VSWM span	0.01	0.03	0.05	0.32	0.793
Naming numbers RT	–0.01	0.02	–0.10	–0.71	0.590
Naming letters RT	0.00	0.02	0.00	0.01	0.992
Digit span	0.01	0.01	0.15	1.84	0.050
NVIQ	–0.00	0.00	–0.07	–0.84	0.480
Subitizing RT	–0.02	0.01	–0.29	–3.83	0.001^∗∗^
**Model 1b – Variability Profiles**					
VSWM span	0.01	0.02	0.04	0.31	0.785
Naming numbers RT	–0.01	0.02	–0.12	–0.88	0.458
Naming letters RT	–0.00	0.02	–0.02	–0.20	0.857
Digit span	0.01	0.01	0.11	1.40	0.130
NVIQ	–0.00	0.00	–0.08	–0.96	0.447
Subitizing RT	–0.01	0.01	–0.19	–2.37	0.023^∗^
Typical Pathway^a^	0.12	0.04	0.39	2.63	0.036^∗^
Delayed Pathway^a^	0.03	0.04	0.11	0.81	0.550

*^*a*^Dummy-coded relative to the Deficit pathway. *P*-values are bias-corrected accelerated bootstrap estimates (1000 samples). ^∗∗^*p* < 0.01, ^∗^*p* < 0.05.*

**Table 6:** Model 2: Linear regression predicting DDA PRT variability.

	*Beta*	*SE*	*β*	*t*	*p*
**Model 2a – Cognitive abilities**					
VSWM span	–5.21	6.70	–0.10	–0.78	0.506
Naming numbers RT	2.90	4.29	0.09	0.68	0.573
Naming letters RT	0.47	4.86	0.01	0.10	0.913
Digit span	–3.38	1.45	–0.17	–2.33	0.053
NVIQ	1.01	0.94	0.08	1.07	0.388
Subitizing RT	8.27	1.37	0.43	6.02	0.001^∗∗^
**Model 2b – Variability Profiles**					
VSWM span	–5.30	6.13	–0.14	–0.87	0.452
Naming numbers RT	3.71	3.92	0.12	0.95	0.433
Naming letters RT	1.39	4.45	0.02	0.31	0.740
Digit span	–2.29	1.34	–0.12	–1.71	0.153
NVIQ	1.22	0.86	0.10	1.41	0.245
Subitizing RT	5.22	1.39	0.27	3.75	0.003^∗∗^
Typical pathway^a^	–54.56	11.25	–0.64	–4.85	0.001^∗∗^
Delayed pathway^a^	–27.38	10.70	–0.32	–2.56	0.062

*^*a*^Dummy-coded relative to the Deficit pathway. *P*-values are bias-corrected accelerated bootstrap estimates (1000 samples). ^∗∗^*p* < 0.01.*

## Discussion

The study investigated whether different patterns of change in SDA PRT trajectories in primary/elementary aged children could be identified over a 6 years period, and the degree to which these patterns reflect typical, delayed or deficit math acquisition pathways. It also assessed the degree to which different SDA PRT change pathways were associated with differences in VSWM, WM, NVIQ, digit naming and subitizing speed, as well as the degree to which the different SDA PRT pathways predicted double digit mental addition problem solving speed and accuracy.

Four findings are of note. First, three distinctly different SDA PRT pathways were identified. In one, labeled a typical acquisition pathway, mean SDA PRT was relatively fast, with relatively little PRT variability. In the second, labeled a delayed pathway, both SDA PRT means and variability were high initially, but diminished over time. In the third pathway, labeled a deficit pathway, SDA PRT mean and variability remained relatively high over the 6 years assessment period. As noted earlier, nearly all children were able to solve SDA problems correctly. Second, with one exception, the three SDA PRT pathways differed in the subitizing variability measure only, and no other cognitive measures. The exception was WM was associated with DDA problem solving success. Third, the subitizing variability measure remained associated with both the DDA success and variability measures, after the pathway factor had been included in regression equations. Fourth, the typical pathway contributed to the equation predicting DDA variability over and above the deficit pathway; and the delayed pathway over and above the deficit pathway. And, the typical pathway contributed to the equation predicting DDA problem solving success over and above the deficit pathway; however, the delayed pathway did not contribute to the prediction equation over and above the deficit pathway.

The pattern of findings support the claims that (1) speed variability signatures are associated with math problem solving ability, even when problems are solved correctly, (2) with the exception of subtizing speed signatures, standard cognitive indices appear unrelated to SDA speed variability indices; and (3) variability in dot enumeration speed signatures within the subitizing range predicts math ability (at least, double-digit mental addition ability). The question remains, why are dot enumeration speed variability signatures specifically, and problem solving variability signatures generally, a predictor of individual difference in math abilities? One answer to this question lies in understanding the reason(s) for differences in dot enumeration subitizing ability.

In a series of studies, we have shown that dot enumeration abilities, and subitizing ability in particular, are associated with children’s math abilities (Reeve et al., 2012; Gray and Reeve, 2014, 2016; Major et al., 2017). In large measure, these studies were motivated by a desire to better understand the reasons for individual differences in Butterworth’s dot enumeration task (see Butterworth’s, 2003, “Dyscalculia Screener”). Reeve et al. (2012) showed that individual differences in children’s subitizing abilities (indexed by the subitizing range, slope and intercept) assessed at school entry predicted math performance across the primary/elementary school years. While these subitizing indices “improved” across time, children’s performance changed at a relative rate compared to each other (i.e., rank order correlations remain stable). Moreover, Major et al. (2017) showed that subitizing abilities assessed at school entry was as good a predictor of school math performances as performance on a standardized math test (The Test of Early Mathematics Ability) in the short term, and a much better predictor in the long term. Furthermore, Gray and Reeve (2014, 2016) showed that pre-schooler’s dot enumeration abilities also predict their emerging math abilities. Other researchers have also found a relationship between subitizing dot enumeration and poor math abilities (Desoete et al., 2009; Reigosa-Crespo et al., 2012; Landerl, 2013).

These findings indirectly emphasize the importance of variability in subitizing speed as a predictor of math ability, but not the reason(s) for its importance. We suggest that poor subitizing abilities reflect a lack an ability to readily extract pattern or grouping information from small sets of dots (Butterworth, 2003; Ashkenazi et al., 2013). Why might this be important for numerical cognition? The ability to “know” the number “2” or “3” can be represented by a collection of two or three dots respectively, without counting individual dots, is arguably a fundamental index of set knowledge (Butterworth, 2010). In the absence of “automatic” set extraction ability, individuals would need to count individual dots. Indeed, set manipulation ability is argued to be an important ability in the development of numerical cognition (Gallistel and Gelman, 1992). We suggest the three speed profiles identified herein reflect different levels of set extraction ability. In the absence of set knowledge, numerical reasoning is likely to be difficult, as is evident in individuals with developmental dyscalculia, who appear to lack the ability to extract information from small sets of dots at a glance (Butterworth, 2010).

The number of children assigned to the deficit profile in the current analysis (8.5% of the sample) is similar to the number of children thought to possess dyscalculia in general population (see Butterworth, 2010). Insofar as the variability in the speed with which small arrays of dots are enumerated is an index of set ability, it is reasonable to ask whether it is a general cognitive or a number specific constraint. It has long been claimed that processing speed is a proxy measure of intelligence (Coyle et al., 2011; however, see Cepeda et al., 2013). Caution should be exercised, however, in arguing for a general processing speed hypothesis on the basis of our findings for two reasons. First, the focus of our research was variability in the speed with which children solve number problems, rather than speed *per se*. Second, while SDA PRT variability and subitizing RT variability independently contributed to the equation predicting double-digit mental addition (success and response time variability), it is difficult to specify the reason(s) for this independence. The acquisition of math ability comprises different components, the importance of which likely varies with age (Dowker, 2005; Gray and Reeve, 2016). It is possible that effective set abilities in the young facilitate the emergence of other math skills, including SDA abilities.

It is worth noting that the variability in speed with which children named the numbers one to nine and the letter A to J was unrelated to other speed variability measures, which argued against the claim that speed variability is a general cognitive constraint, and rather supports the claim that it is number-specific constraint.

### Limitations of Research

In the present study we examined the variability in SDA problem solving speed. On the basis of our previous research, we are reasonably confident problem solving speed reflects SDA strategy-use—immature SDA strategies take longer to execute than more mature strategies (see Canobi et al., 1998, 2002; Paul and Reeve, 2016). Nevertheless, we did not examine the mix of SDA problem solving strategies, or how this mix changes in the typical, delayed and deficit groups over time. It is possible that the speed variability measure may obscure other indices (e.g., variability in speed taken to execute the same SDA strategy over time).

While we have argued for a distinction between a typical, delayed, and deficit math pathway, it is important not to overstate the robustness of this argument for two reasons. First, we have focused on a relatively narrow range of computation abilities (SDA and DDA) over a relatively short time. It is possible, with time, the performance of children in the deficit and delayed groups would approach the performance of children in the typical pathway group. Second, although we focused on mental addition in the pre-adolescent years because of its importance in math curricula, we recognize the pattern of findings may differ for other math competencies (e.g., subtraction, multiplication, division).

## Conclusion

We have argued that the variability in the speed with which children enumerate one to three dots is an index of the ability to rapidly extract set knowledge, which, in turn, is a key ingredient in the acquisition of preadolescent children’s math ability. However, the degree to which set knowledge changes in childhood is yet to be specified precisely, or the degree to which it is supported by other cognitive functions (e.g., attention abilities). Nevertheless, we suggest our findings have diagnostic and intervention implications. Given the variability in dot enumeration RTs is a diagnostic measure of math ability, it is a relatively easy measure to collect and interpret.

## Ethics Statement

This study was conducted in accordance with the recommendations of the Human Ethics Committee of the University of Melbourne. Written informed consent was obtained from the parents of the children who participated in the study. All participants provide informed consent in accordance with the Declaration of Helsinki. The interview protocols were approved by the Human Research Ethics Committee of the University of Melbourne and by participating schools.

## Author Contributions

All authors listed have made a substantial, direct and intellectual contribution to the work, and approved it for publication.

## Conflict of Interest Statement

The authors declare that the research was conducted in the absence of any commercial or financial relationships that could be construed as a potential conflict of interest.
