# Supplementary material for: Variability in Single Digit Addition Problem-Solving Speed Over Time Identifies Typical, Delay and Deficit Math Pathways
Source: Front Psychol. 2018 Aug 14;9:1498. doi: 10.3389/fpsyg.2018.01498 (PMC6102488; doi:10.3389/fpsyg.2018.01498)
Supplement: Supplementary file 1 [file Table_1.DOCX]

**Supplementary Materials for *Variability in Single Digit Addition Problem-solving Speed over Time Identifies Typical, Delay and Deficit Math Pathways***

**Selection of profiles**

As reported in text, while the four-profile solution provided better fit than the three-profile solution (i.e., significant bootstrap likelihood-ratio test scores; see Table S1 reproduced below), examination of the four profiles revealed two profiles were similar—both profiles showed patterns of delayed decrease in variability over time, which were not meaningfully different from each other (see Figure S1).

The three-profile solution characterized more distinct patterns of change in variability over time, and were more consistent with typical, delayed and deficit pathways (see Figure 1). Based on these findings, the three-profile model was selected for further examination.

**Table S1. Latent profile analysis goodness-of-fit indices**

| **Profiles** | **Parameters** | **LL** | **AIC** | **BIC** | **aBIC** | **BLRT** | **Entropy** |
| --- | --- | --- | --- | --- | --- | --- | --- |
| 2 | 17 | -2425.42 | 4884.83 | 4937.53 | 4883.71 | <.001 | 0.86 |
| 3 | 26 | -2373.91 | 4799.83 | 4880.42 | 4798.11 | <.001 | 0.83 |
| 4 | 35 | -2342.39 | 4754.78 | 4863.28 | 4752.47 | <.001 | 0.85 |

Note: LL = Log-likelihood; AIC = Akaike Information Criterion; BIC = Bayesian Information Criterion; aBIC = Adjusted Bayesian Information Criterion; BLRT = Bootstrap Likelihood-Ratio Test (100 draws).
