# Supplementary material for: Variability in Single Digit Addition Problem-Solving Speed Over Time Identifies Typical, Delay and Deficit Math Pathways
Source: Front Psychol. 2018 Aug 14;9:1498. doi: 10.3389/fpsyg.2018.01498 (PMC6102488; doi:10.3389/fpsyg.2018.01498)
Supplement: Supplementary file 2 [file Table_2.DOCX]

**Supplementary Materials for *Variability in Single Digit Addition Problem-solving Speed over Time Identifies Typical, Delay and Deficit Math Pathways***

**Average latent profile probabilities for most likely latent profile membership**

Aside from the high entropy value for the three-profile solution (indicating good separability of profiles), the average latent profile probabilities for most likely latent profile membership were high for each profile (see Table S2). Since these values were high, children were allocated to the profile with their highest likelihood of membership for subsequent analyses.

**Table S2. Average latent profile probabilities for most likely latent profile membership**

|  | Typical | Delayed | Deficit |
| --- | --- | --- | --- |
| Typical | 0.936 | 0.064 | 0.000 |
| Delayed | 0.043 | 0.933 | 0.025 |
| Deficit | 0.000 | 0.077 | 0.925 |
